# Cognitive Diagnostic Models for Rater Effects

**DOI:** 10.3389/fpsyg.2020.00525

**Published:** 2020-03-24

**Authors:** Xiaomin Li, Wen-Chung Wang, Qin Xie

**Affiliations:** ^1^Centre for Child and Family Science, The Education University of Hong Kong, Tai Po, Hong Kong; ^2^Assessment Research Centre, The Education University of Hong Kong, Tai Po, Hong Kong; ^3^Department of Linguistics and Modern Language Studies, The Education University of Hong Kong, Tai Po, Hong Kong

**Keywords:** cognitive diagnostic models, facets models, hierarchical rater models, rater effect, item response theory

## Abstract

In recent decades, cognitive diagnostic models (CDMs) have been intensively researched and applied to various educational and psychological tests. However, because existing CDMs fail to consider rater effects, the application of CDMs to constructed-response (CR) items that involve human raters is seriously limited. Given the popularity of CR items, it is desirable to develop new CDMs that are capable of describing and estimating rater effects on CR items. In this study, we developed such new CDMs within the frameworks of facets models and hierarchical rater models, using the log-linear cognitive diagnosis model as a template. The parameters of the new models were estimated with the Markov chain Monte Carlo methods implemented in the freeware JAGS. Simulations were conducted to evaluate the parameter recovery of the new models. Results showed that the parameters were recovered fairly well and the more data there were, the better the recovery. Implications and applications of the new models were illustrated with an empirical study that adopted a fine-grained checklist to assess English academic essays.

In the past few decades, extensive research has been conducted in the area of cognitive diagnosis, and a wide range of cognitive diagnostic models (CDMs) (also called diagnostic classification models; DCMs) has been developed to provide fine-grained information about students’ learning strengths and weaknesses (Tatsuoka, 1985; Templin, 2004; de la Torre, 2011; Chiu and Douglas, 2013; Hansen and Cai, 2013). Popular CDMs include the deterministic inputs, noisy and gate (DINA) model (Haertel, 1989; Junker and Sijtsma, 2001; de la Torre and Douglas, 2004), the deterministic input, noisy or gate model (Templin and Henson, 2006), and the reduced reparameterized unified model (Hartz, 2002). Unlike unidimensional item response theory (IRT), which provides a single score for a student’s proficiency on a latent continuum, CDMs offer a profile of multiple binary (mastery or non-mastery) statuses of certain knowledge or skills.

In applications of CDMs, item responses to multiple-choice items, for example, are assumed to be objectively scored. In many situations, such as educational assessment, performance appraisal, psychological diagnosis, medical examination, sports competition, and singing contests, responses to constructed-response (CR) or performance-based items are evaluated by human raters. Different raters often exhibit different degrees of severity. There are two major approaches to rater effects in the IRT framework. One is to treat raters as a third facet, in addition to the item and person facets, to highlight the impact of rater effects on the item scores. Examples are the Rasch facets models (Linacre, 1989) and the random-effect facets model (Wang and Wilson, 2005). The other approach is to employ signal detection theory to describe raters’ judgment. Examples include the hierarchical rater model (HRM; Patz et al., 2002) and the latent class extension of signal detection theory (DeCarlo et al., 2011). The facets approach and the HRM approach have very different assumptions regarding rater behaviors, as discussed below. The resulting measures of a person (ratee) can only be considered fair and valid for individual comparison if rater effects are directly accounted for in the IRT models.

Rater effects can happen in the CDM framework when raters are recruited to mark item responses. In this study, we adapt these two approaches (facets and HRM) to the CDM framework to account for rater effects. Based on the same logic above, the resulting profiles (a set of binary latent attributes) of persons (ratees) are fair and valid for individual comparison only when rater effects are directly accounted for in the CDMs. The remainder of this paper is organized as follows. First, the facets and HRM approaches within the IRT framework are briefly introduced. Second, these two approaches are adapted to the CDM framework to create new CDMs to account for rater effects. Third, a series of simulations are conducted to evaluate the parameter recovery of the new CDMs, and their results are summarized. Fourth, an empirical example about essay writing is provided to demonstrate applications of the new models. Finally, conclusions are drawn and suggestions for future studies are provided.

## Introduction to the Facets and HRM Approaches

### The Facets Approach

In the facets approach, raters are treated as instruments to measure ratees, just like items are. Raters are recruited to provide their own expertise to make judgments of ratees’ performance; therefore, the more raters there are, the more reliable the measurement of the ratees. In the facets model (Linacre, 1989), the log-odds (logit) of scoring *k* over *k* – 1 on item *j* for ratee *i* judged by rater *r* is defined as:

(1) $$\log\left(P_{\text{ijkr}}/P_{\mathrm{ij}\,\left(k-1\right)\,r}\right)=\theta_{\text{i}}-\beta_{\text{jk}}-\eta_{\text{r}}$$

where *P*_ijkr_ and *P*_ij(k–1)r_ are the probabilities of receiving a score of *k* and *k* - 1, respectively, for ratee *i* on item *j* from rater *r*; θ_i_ is the latent (continuous) trait of ratee *i* and is often assumed to follow a normal distribution; β_jk_ is the *k*th threshold of item *j*; η_r_ is the severity of rater *r*. A positive (negative) η_r_ decreases (increases) the probability of receiving a high score. Equation 1 can be easily generalized to more than three facets.

In Equation 1, a rater has a single parameter η_r_ to account for the rater’s degree of severity, meaning that the rater holds a constant degree of severity throughout all ratings. In reality, it is likely that a rater exhibits some fluctuations in severity when giving ratings. If so, Equation 1 is too stringent, and the assumption of constant severity needs to be relaxed. To account for the intra-rater fluctuations in severity, Wang and Wilson (2005) proposed adding a random-effect parameter to the facets model, which can be expressed as:

(2) $$\log\,\left(P_{\text{ijkr}}/P_{\mathrm{ij}\,\left(k-1\right)\,r}\right)=\theta_{\text{i}}-\beta_{\text{jk}}-\left(\eta_{\text{r}}+\zeta_{\text{ir}}\right)$$

where ζ_ir_ is assumed to follow a normal distribution, with mean 0 and variance $\sigma_{\text{r}}^{2}$; others have been defined in Equation 1; θ and ζ are assumed to be mutually independent. Where appropriate, slope parameters can be added and covariates (e.g., gender) can be incorporated to account for variations in θ and η (Wang and Liu, 2007). The facets models have been widely used to account for rater effects in practice (Engelhard, 1994, 1996; Myford and Wolfe, 2003, 2004).

### The HRM Approach

In the HRM approach, it is argued that thorough scoring rubrics can (in theory) be programmed into computers so human raters are no longer needed (Patz et al., 2002). However, until computer scoring is made possible (e.g., it is not cost-effective to develop e-raters), human raters are still in demand but they are expected to function like scoring machines (clones) as closely as possible. Unfortunately, human judgment may deviate remarkably from machine scoring, which brings random noise to the ratings. Only when raters act exactly like scoring machines will a CR item provide as much information as an objective (machine-scorable) item does. Following this logic, increasing the number of raters will not increase the precision of ratee measurements.

The HRM involves two steps. In the first step, the scores provided by raters are treated as indicators of the latent (true, or ideal) category for ratee *i*’s response to item *j*. Let ξ_ij_ be the latent category for ratee *i* on item *j*. The probability that rater *r* will assign a rating *k* given ξ_ij_ is assumed to be proportional to a normal density with a mean ξ_ij_ – ϕ_r_ and a standard deviation ψ_r_:

(3) $$P_{\text{ijkr}}\propto \exp\left[-\frac{1}{2\,\psi_{\text{r}}^{2}}\,{\left[k-\left(\xi_{\text{ij}}-\phi_{\text{r}}\right)\right]}^{2}\right]$$

where ϕ_r_ represents the severity for rater *r*: a value of 0 indicates the rater is most likely to provide the same rating as the latent (true) category, a negative value indicates that the rater tends to be lenient, a positive value implies that the rater tends to be severe, and ψ_r_ represents the rater’s variability: the larger the value, the less reliable (consistent) the ratings.

In the second step, the latent category ξ_ij_ is used as the indicator of a ratee’s ability via an IRT model such as the partial credit model (Masters, 1982):

(4) $$P_{\text{ijl}}\equiv P\left(\xi_{\text{ij}}=l|\theta_{\text{i}}\right)=\frac{\exp\,\sum_{k=0}^{\text{l}}\left(\theta_{\text{i}}-\delta_{\text{jk}}\right)}{\sum_{m=0}^{{\text{M}}_{\text{j}}}\text{exp}\,\sum_{k=0}^{\text{m}}\left(\theta_{\text{i}}-\delta_{\text{jk}}\right)}$$

(5) $$\text{logit}\,\left(P_{\text{ijl}}\right)\equiv \log\left(P_{\text{ijl}}/P_{\mathrm{ij}\,\left(l-1\right)}\right)=\theta_{\text{i}}-\delta_{\text{jk}}$$

where *M*_j_ is the maximum score of item *j*, δ_jk_ is the *k*th step parameter of item *j*, θ_i_ is the latent trait for person *i*. By defining $\sum_{k=0}^{0}\left(\theta_{\text{i}}-\delta_{\text{jk}}\right)\equiv 0$ and $\sum_{k=0}^{\text{m}}\left(\theta_{\text{i}}-\delta_{\text{jk}}\right)\equiv \sum_{k=1}^{\text{m}}\left(\theta_{\text{i}}-\delta_{\text{jk}}\right)$, the probability of scoring 0 is $P_{\mathrm{ij0}}=\frac{1}{\sum_{m=0}^{M_{\text{j}}}\text{exp}\,\sum_{k=0}^{\text{m}}\left(\theta_{\text{i}}-\delta_{\text{jk}}\right)}$. Note that ξ_ij_ in Equation 4 is latent rather than observed in the standard partial credit model.

A problem in the HRM, also noted by Patz et al. (2002), is that a relatively small value for ψ_r_ would lead to difficulties in determining a unique value for ϕ_r_ because the posterior distribution of ϕ_r_ is almost uniform (DeCarlo et al., 2011). Another limitation of the HRM is that it can account for a rater’s severity and inconsistency, but not for other rater effects, such as centrality. To resolve these problems, DeCarlo et al. (2011) extended the HRM by incorporating a latent class extension of the signal detection theory as:

(6) $$P_{{\mathrm{ijk}}^{*}\,r}=F\,\left[a_{\text{jr}}\,\left(\xi_{\text{ij}}-c_{\text{jkr}}\right)\right],$$

where *P*_ijk*r_ denotes the probability of assigning a rating less than or equal to *k* (denoted as *k*^∗^) given ξ_ij_; *F* can be a cumulative normal or logistic distribution; *a*_jr_ is a slope (sensitivity) parameter for rater *r* on item *j*; *c*_jkr_ is the *k*th ordered location parameter of item *j* for rater *r*. Like ψ_r_ in Equation 3, *a*_jr_ depicts how sensitive or reliable the ratings are for rater *r* on item *j*. A close investigation of *c*_jkr_ can reveal rater severity and centrality. Further, by including an autoregressive time series process and a parameter for overall growth, the HRM approach is also feasible for longitudinal data (Casabianca et al., 2017).

## The Log-Linear Cognitive Diagnosis Model

Cognitive diagnostic models have been applied to large-scale educational assessments such as the Trends in International Mathematics and Science Study (TIMSS), the Progress in International Reading Literacy Study (PIRLS), the National Assessment of Educational Progress (NEAP), and the Test of English as a Foreign Language (TOEFL) to obtain information about students’ cognitive abilities (Tatsuoka et al., 2004; Xu and von Davier, 2008; Chiu and Seo, 2009; Templin and Bradshaw, 2013). In these datasets, both multiple-choice items and CR items are used. For example, in the PIRLS reading comprehension test, approximately half of the items require examinees to write down their responses, which are then marked by human raters. In these studies of fitting CDMs to large-scale educational assessments, rater effects were not considered simply because existing CDMs could not account for rater effects. To resolve this problem, we developed new CDMs for rater effects within both the facets and HRM frameworks. We adopted the log-linear cognitive diagnosis model (LCDM; Henson et al., 2009) as a template because it includes many CDMs as special cases. Nevertheless, the new models developed in this study can also apply easily to other general CDMs, such as the general diagnostic model (von Davier, 2008) or the generalized DINA model (de la Torre, 2011).

Under the LCDM, the probability of success (scoring 1) on item *j* for person *i* is defined as:

(7) $$P_{\mathrm{ij1}}\equiv P\left(X_{\text{ij}}=1|\alpha_{\text{i}}\right)=\frac{\exp\left(\lambda_{j,0}+\lambda_{\text{j}}^{\text{T}}\,h\,\left(\alpha_{\text{i}},{\text{q}}_{\text{j}}\right)\right)}{1+\exp\left(\lambda_{j,0}+\lambda_{\text{j}}^{\text{T}}\,h\,\left(\alpha_{\text{i}},{\text{q}}_{\text{j}}\right)\right)}$$

(8) $$\text{logit}\,\left(P_{\mathrm{ij1}}\right)\equiv \log\left[P_{\mathrm{ij1}}/\left(1-P_{\mathrm{ij1}}\right)\right]=\lambda_{j,0}+\lambda_{\text{j}}^{\text{T}}\,h\,\left(\alpha_{\text{i}},{\text{q}}_{\text{j}}\right)$$

where α_i_ is the latent profile of person *i*, λ_j,__0_ defines the probability of success for those persons who have not mastered any of the attributes required by item *j*; $\lambda_{\text{j}}^{\text{T}}$ is a (2^*K*^−1) by 1 vector of weights for item *j*; *q*_jk_ is the entry for item *j* in the Q-matrix; *h*(α_i_,**q**_j_) is a set of linear combinations of α_*i*_ and **q**_j_; $\lambda_{\text{j}}^{\text{T}}\,h\,\left(\alpha_{\text{i}},{\text{q}}_{\text{j}}\right)$ can be written as:

(9) λjTh(αi,q)j=∑k=1Kλjk(αikqjk)+∑k=1K∑v>kλjkv(αikαivqjkqjv)+…

For item *j*, the exponent includes an intercept term, all main effects of attributes, and all possible interaction effects between attributes. By constraining some of the LCDM parameters, many existing CDMs can be formed (Henson et al., 2009). For example, for a three-attribute item, the DINA model can be defined as:

(10) $$P_{\mathrm{ij1}}=\frac{\exp\left(\lambda_{j,0}+\lambda_{j,123}\,\alpha_{\mathrm{i1}}\,\alpha_{\mathrm{i2}}\,\alpha_{\mathrm{i3}}\right)}{1+\exp\left(\lambda_{j,0}+\lambda_{j,123}\,\alpha_{\mathrm{i1}}\,\alpha_{\mathrm{i2}}\,\alpha_{\mathrm{i3}}\right)}$$

Although we concentrate on dichotomous responses in this study for illustrative purpose, Equation 7 can be extended to accommodate polytomous items. Let *P*_ijk_ and *P*_ij(__k__–__1__)_ be the probabilities of scoring *k* and *k* − 1 on item *j* for person *i*, respectively. Equation 8 can be extended as:

(11) $$\text{logit}\,\left(P_{\text{ijk}}\right)\equiv \log\left(P_{\text{ijk}}/P_{\mathrm{ij}\,\left(k-1\right)}\right)=\lambda_{j,0,k-1}+\lambda_{\text{j}}^{\text{T}}\,h\,\left(\alpha_{\text{i}},{\text{q}}_{\text{j}}\right),$$

where λ_j,0,k–1_ is the (*k* – 1)th intercept for item *j*. Equation 11 is based on adjacent-category logit. Actually, cumulative logit (Hansen, 2013) and other approaches are also feasible (Ma and de la Torre, 2016).

For the ease of understanding and interpretation, item parameters in the LCDM can be expressed as follows, which is commonly called as the guessing parameters (*g*_j_) and slip parameters (*s*_j_):

(12) $$g_{\text{j}}=\frac{\exp\left(\lambda_{j,0}\right)}{1+\exp\left(\lambda_{j,0}\right)}$$

(13) $$s_{\text{j}}=1-\frac{\exp\left(\lambda_{j,0}+\lambda_{\text{j}}^{\text{T}}\,\left(\alpha_{\text{i}},{\text{q}}_{\text{j}}\right)\right)}{1+\exp\left(\lambda_{j,0}+\lambda_{\text{j}}^{\text{T}}\,\left(\alpha_{\text{i}},{\text{q}}_{\text{j}}\right)\right)}$$

representing the probability of success without mastering all the required attributes, and the probability of failure with mastering all the required attributes, respectively.

## New CDMs With the Facets Approach

All existing CDMs involve two facets: person and item. When items are marked by human raters, a third facet is needed to account for rater effects. To accomplish this, Equation 8 can be extended as:

(14) $$\text{logit}\,\left(P_{\mathrm{ijr1}}\right)\equiv \log\left[P_{\mathrm{ijr1}}/\left(1-P_{\mathrm{ijr1}}\right)\right]=\lambda_{j,0}-\eta_{\text{r}}+\lambda_{\text{i}}^{\text{T}}\,h\,\left(\alpha_{\text{i}},{\text{q}}_{\text{j}}\right)$$

where *P*_ijr__1_ is the probability of success (scoring 1) on item *j* for person *i* marked by rater *r*; η_r_ is the severity of rater *r*; other terms have been defined. A positive (negative) η_r_ decreases (increases) the probability of success. If η_r_ = 0 for all raters, Equation 14 simplifies to Equation 8. That is, Equation 14 is a three-facet extension of the LCDM.

When there is a concern about intra-rater variations in severity, η_r_ in Equation 14 can be replaced with η_r_ + ζ_ir_. Moreover, Equation 14 can be easily generalized to include more than three facets. For example, in the Test of Spoken English (TSE) assessment system, examinees’ speaking tasks are marked on multiple criteria by human raters, so four facets are involved: ratee, task, rater, and criterion. In such cases, Equation 14 can be extended to four facets as:

(15) $$\begin{array}{c} \text{logit}\,\left(P_{\mathrm{ijrs1}}\right)\equiv \log\left[P_{\mathrm{ijrs1}}/\left(1-P_{\mathrm{ijrs1}}\right)\right]\\ \;=\lambda_{j,0}-\eta_{\text{r}}-\gamma_{\text{s}}+\lambda_{\text{i}}^{\text{T}}\,h\,\left(\alpha_{\text{i}},{\text{q}}_{\text{j}}\right) \end{array}$$

where *P*_ijrs__1_ is the probability of success (scoring 1) on task *j* along criterion *s* for examinee *i* marked by rater *r*; γ_s_ is the threshold of criterion *s*; other terms have been defined. Generalization to more facets is straightforward. For polytomous items, Equation 14 can be extended as:

(16) $$\text{logit}\,\left(P_{\text{ijrk}}\right)\equiv \log\left(P_{\text{ijrk}}/P_{\mathrm{ijr}\,\left(k-1\right)}\right)=\lambda_{j,0,k-1}-\eta_{\text{r}}+\lambda_{\text{i}}^{\text{T}}\,h\,\left(\alpha_{\text{i}},{\text{q}}_{\text{j}}\right)$$

where *P*_ijr__k_ and *P*_ijr(__k__–__1__)_ be the probabilities of scoring *k* and *k −* 1 on item *j* for examinee *i* marked by rater *r*, respectively; other terms have been defined.

## New CDMs With the HRM Approach

The signal detection model in the first step in the HRM approach can be defined as in Equation 3 or 6, with the constraint of *k* = 0 or 1 because of dichotomous items. For dichotomous items, Equations 3 and 6 become equivalent, except there is a single ψ_r_ for each rater in Equation 3, but multiple *a*_jr_ (across items) for each rater in Equation 6. The IRT model in the second step (Equation 4 or 5) can be replaced with a CDM like the LCDM. Using the LCDM as template, the new model can be written as:

(17) $$P_{\mathrm{ij1}}\equiv P\left(\xi_{i\,\text{j}}=1|\alpha_{\text{i}}\right)=\frac{\exp\left(\lambda_{j,0}+\lambda_{\text{j}}^{\text{T}}\,h\,\left(\alpha_{\text{i}},{\text{q}}_{\text{j}}\right)\right)}{1+\exp\left(\lambda_{j,0}+\lambda_{\text{j}}^{\text{T}}\,h\,\left(\alpha_{\text{i}},{\text{q}}_{\text{j}}\right)\right)}$$

(18) $$\text{logit}\,\left(P_{\mathrm{ij1}}\right)\equiv \log\left[P_{\mathrm{ij1}}/\left(1-P_{\mathrm{ij1}}\right)\right]=\lambda_{j,0}+\lambda_{\text{j}}^{\text{T}}\,h\,\left(\alpha_{\text{i}},{\text{q}}_{\text{j}}\right)$$

where ξ_ij_ is the latent binary category of person *i* on item *j*; other terms have been defined. Comparing Equations 17 and 7, one finds that the category is latent in Equation 17, but observed in Equation 7. For polytomous items, Equation 18 can be extended as:

(19) $$\text{logit}\,\left(P_{\text{ijk}}\right)\equiv \log\left(P_{\text{ijk}}/P_{\mathrm{ij}\,\left(k-1\right)}\right)=\lambda_{j,0,k-1}+\lambda_{\text{j}}^{\text{T}}\,h\,\left(\alpha_{\text{i}},{\text{q}}_{\text{j}}\right)$$

## Parameter Estimation

Parameters in the new facets-CDM and HRM-CDMs can be estimated by utilizing Markov chain Monte Carlo (MCMC) methods (de la Torre and Douglas, 2004; Ayers et al., 2013), which treat parameters as random variables and repeatedly draw from their full conditional posterior distributions over a large number of iterations. In this study, the freeware JAGS (Version 4.2.0; Plummer, 2015) and the R2jags package (Version 0.5-7; Su and Yajima, 2015) in *R* (Version 3.3.0 64-bit; R Core Team, 2016) were used to estimate model parameters. JAGS uses a default option of the Gibbs sampler and offers a user-friendly tool for constructing Markov chains for parameters, so the derivation of the joint posterior distribution of the model parameters becomes attainable. We used the Gelman–Rubin diagnostic statistic (Gelman and Rubin, 1992) to assess convergence, in which a value smaller than 1.1 is typically regarded as convergence as a rule of thumb. In the facets-CDMs, the rater severity was constrained at a zero mean for model identification. Our pilot simulation supported the use of 10,000 iterations, with the first 5,000 iterations as burn-in and the remaining 5,000 iterations for the point estimates (expected *a posteriori*) and their standard errors by sampling one in every 10 values. The resulting Gelman–Rubin diagnostic statistic indicated no convergence problem.

Two simulation studies were conducted to evaluate the recovery of item parameters and person profiles for the two newly proposed models with rater effects. Moreover, we evaluated the effects of ignoring rater effects by comparing the proposed models (with rater effect) and standard models (without rater effect) in the simulations. In particular, Study I evaluated the item and person recovery of the facets-CDM under different rating designs. Study II assessed the implementation of the HRM-CDM. One hundred replications were conducted under each condition. For comparison, all simulated data were also analyzed with the standard CDMs, which did not consider rater effects.

## Simulation Study I: Facets-CDM

### Design

Rating design is a practical issue because it involves resource allocation. A good rating design can save a great deal of resource while holding acceptable precision of ratee measurement. According to the procedures of Chiu et al. (2009), latent ability θ of 500 ratees were drawn from a multivariate normal distribution *MVN*(0,Σ), with the diagonal and off-diagonal elements of the covariance matrix taking a value of 1 and 0.5, respectively. A correlation of 0.5 between attributes was specified to mimic moderate to medium correlations between attributes in educational settings. Assuming that the underlying continuous ability for the *i*th ratee was $\theta_{\text{i}}^{\text{T}}=\left(\theta_{\mathrm{i1}},\theta_{\mathrm{i2},}\,\cdots ,\theta_{\text{iK}}\right)$, the profile pattern $\alpha_{\text{i}}^{\text{T}}=\left(\alpha_{\mathrm{i1}},\alpha_{\mathrm{i2}},\cdots ,\alpha_{\text{iK}}\right)$ was determined by

(20) $$\alpha_{\text{ik}}=\left\{ \begin{array}{c} 1,\mathrm{if}\,\theta_{\text{ik}}\geq \Phi^{-1}\,\left(\frac{k}{K+1}\right),\\ 0,\mathrm{otherwise}. \end{array}\right.$$

The test consisted of 10 dichotomous items measuring five attributes, as shown in Table 1, and 10 raters. Dichotomous responses were simulated according to the facets-CDM (Equation 11). The generating intercepts (λ_j,0_), main effects (λ_j,1_), two-way interactions (λ_j,2_), and rater severities (η_r_) are listed in Table 3, and the resulting range of the guessing parameters and slip parameters was [0.08, 0.20] and [0.07, 0.19], respectively.

**TABLE 1:** Q-matrix for the ten items in the simulations.

Item	Attribute 1	Attribute 2	Attribute 3	Attribute 4	Attribute 5
1	1	0	0	0	0
2	0	1	0	0	0
3	0	0	1	0	0
4	0	0	0	1	0
5	0	0	0	0	1
6	1	1	0	0	0
7	0	1	1	0	0
8	0	0	1	1	0
9	0	0	0	1	1
10	1	0	0	0	1

*1s mean the attributes are required, and 0s mean the attributes are not required.*

Four kinds of rating design were used: (a) completely crossed design, where every ratee was judged by every rater; (b) balanced incomplete design, where each ratee was judged by three raters and each rater judged 150 ratees; (c) unbalanced incomplete design, where each ratee was judged by three raters but different raters judged different numbers of ratees; (d) random design, where 20 ratees were judged by all raters and the remaining 480 ratees were judged by three raters randomly selected from the rater pool. The completely crossed design, although seldom used when there are a large number of ratees (e.g., several hundred), was adopted here to provide reference information about the parameter recovery of the facets-CDMs. In the three incomplete designs, raters were connected by a set of common ratees. Detailed specification of the incomplete designs is shown in Table 2.

**TABLE 2:** Number of ratees under the incomplete designs in simulation study I (Facets-CDM).

Rater										
	**1**	**2**	**3**	**4**	**5**	**6**	**7**	**8**	**9**	**10**
**Balanced**										
	50	50	50							
		50	50	50						
			50	50	50					
				50	50	50				
					50	50	50			
						50	50	50		
							50	50	50	
								50	50	50
	50								50	50
	50	50								50
Total	150	150	150	150	150	150	150	150	150	150
**Unbalanced**										
	50	50	50							
		68	68	68						
			44	44	44					
				58	58	58				
					35	35	35			
						51	51	51		
							50	50	50	
								55	55	55
	40								40	40
	49	49								49
Total	139	167	162	170	137	144	136	156	145	144
**Random**										
Total	134	155	157	141	168	158	152	130	153	152

### Analysis

The generated data were analyzed with (a) the data-generating facets-CDM (saturated) model and (b) the standard CDM without considering rater effects, where the ratings given by the raters were treated as responses to virtual items with identical item parameters. Based on prior studies (e.g., Li and Wang, 2015; Zhan et al., 2019), a less informative normal prior was specified for all model parameters across the two models. Specifically, a normal prior with mean zero and standard deviation four was assumed for the intercepts (λ_j,0_), main effects (λ_j,1_), two-way interactions (λ_j,2_), and rater severities (η_r_). Moreover, a truncated normal distribution was specified to constraint the main effect parameters (λ_j,1_) to be positive. In doing so, the probabilities of correct responses increased as a function of mastering each required attribute. To evaluate the recovery of item parameters, we computed the bias and root mean squared error (RMSE) of these estimates across replications. For person profiles, we computed the mean accurate recovery rate. In the completely crossed design, each item received 5,000 scores (500 ratees times 10 raters), each ratee received 100 scores (10 items times 10 raters), and each rater gave 5,000 scores (10 items times 500 ratees); in the three incomplete designs, each item received approximately 1,500 scores (500 ratees times 3 raters), each ratee received 30 scores (10 items times 3 raters) except 20 ratees received 100 scores (10 items times 10 raters) in the random design, and each rater gave approximately 1,500 scores (10 items times 150 ratees). In general, the more the data points, the better the parameter estimation and profile recovery. It was thus anticipated that when the facets-CDM was fit, the parameter estimation and recovery rates would be better in the completely crossed design than in the three incomplete designs. When the standard CDM was fit, the parameter estimation and recovery rates would be poor because the rater effects were not considered.

### Results

Table 3 lists the generating values, the bias values, and the RMSE values for the two models under the four designs. When the facets-CDM was fit, the RMSE values were not large, ranging from 0.07 to 0.24 (*M* = 0.16) in the completely crossed design, from 0.10 to 0.52 (*M* = 0.23) in the balanced incomplete design, from 0.12 to 0.51 (*M* = 0.23) in the unbalanced incomplete design, and from 0.10 to 0.49 (*M* = 0.22) in the random design. Such small RMSE values suggested good parameter recovery and they were similar to those found in common CDMs (e.g., De la Torre et al., 2010; Huang and Wang, 2014). With respect to the recovery of the latent profile, the mean recovery rate across profiles was 97.58% in the completely crossed design, 71.02% in the balanced incomplete design, 70.14% in the unbalanced incomplete design, and 72.62% in the random design. As expected, the parameter estimation and profile recovery were better in the completely crossed design than in the incomplete designs.

**TABLE 3:** Generating values, bias, root mean square error (RMSE), and profile recovery rates (%) in simulation study I (Facets-CDM).

		Complete design				Balanced design				Unbalanced design				Random design			
		Facets-CDM		Standard CDM		Facets-CDM		Standard CDM		Facets-CDM		Standard CDM		Facets-CDM		Standard CDM	
Par.	Gen	Bias	RMSE	Bias	RMSE	Bias	RMSE	Bias	RMSE	Bias	RMSE	Bias	RMSE	Bias	RMSE	Bias	RMSE
λ_1,0_	–2.00	–0.13	0.21	0.30	0.30	–0.21	0.28	0.08	0.11	–0.12	0.22	0.16	0.19	–0.12	0.22	0.17	0.19
λ_2,0_	–1.40	–0.12	0.19	0.23	0.23	–0.19	0.25	0.04	0.07	–0.21	0.25	–0.02	0.06	–0.13	0.18	0.11	0.13
λ_3,0_	–1.79	–0.13	0.20	0.27	0.27	–0.18	0.25	0.09	0.16	–0.18	0.24	0.09	0.13	–0.20	0.25	0.10	0.14
λ_4,0_	–1.37	–0.15	0.21	0.20	0.20	–0.19	0.26	0.02	0.09	–0.17	0.23	0.08	0.09	–0.21	0.28	0.03	0.08
λ_5,0_	–1.85	–0.12	0.21	0.28	0.28	–0.28	0.31	0.01	0.09	–0.16	0.21	0.10	0.15	–0.16	0.19	0.17	0.20
λ_6,0_	–2.42	–0.14	0.23	0.33	0.33	–0.26	0.33	–0.06	0.16	–0.11	0.18	–0.05	0.18	–0.30	0.36	–0.05	0.13
λ_7,0_	–1.57	–0.10	0.20	0.26	0.27	–0.12	0.22	0.01	0.16	–0.09	0.29	–0.03	0.16	–0.11	0.24	0.07	0.10
λ_8,0_	–1.95	–0.10	0.21	0.31	0.32	–0.17	0.24	0.00	0.14	–0.18	0.23	–0.02	0.17	–0.19	0.24	–0.02	0.18
λ_9,0_	–2.07	–0.11	0.20	0.32	0.33	–0.22	0.31	–0.06	0.23	–0.09	0.18	0.03	0.13	–0.12	0.27	0.07	0.21
λ_10,0_	–1.69	–0.10	0.22	0.28	0.29	–0.18	0.28	–0.05	0.12	–0.08	0.29	0.02	0.16	–0.09	0.17	0.09	0.12
λ_1,1_	3.72	–0.01	0.09	–0.62	0.62	–0.03	0.20	–0.63	0.65	–0.13	0.18	–0.84	0.84	–0.03	0.14	–0.60	0.62
λ_2,1_	2.82	–0.06	0.10	–0.55	0.55	–0.15	0.21	–0.56	0.58	–0.19	0.24	–0.58	0.59	–0.18	0.20	–0.65	0.65
λ_3,1_	3.41	0.03	0.08	–0.55	0.56	–0.01	0.18	–0.58	0.60	–0.05	0.17	–0.61	0.62	–0.05	0.11	–0.67	0.68
λ_4,1_	2.91	0.01	0.08	–0.50	0.50	–0.06	0.10	–0.42	0.43	–0.09	0.14	–0.58	0.59	–0.04	0.18	–0.50	0.52
λ_5,1_	3.38	0.00	0.10	–0.56	0.57	–0.05	0.10	–0.53	0.54	–0.03	0.12	–0.59	0.60	–0.08	0.14	–0.63	0.65
λ_6,1_	1.26	0.04	0.10	–0.12	0.14	–0.02	0.18	0.02	0.16	–0.13	0.20	0.04	0.15	0.14	0.29	0.13	0.27
λ_7,1_	1.04	–0.04	0.07	–0.20	0.20	–0.03	0.26	–0.01	0.21	–0.15	0.28	–0.04	0.19	–0.09	0.20	–0.10	0.17
λ_8,1_	1.18	–0.05	0.09	–0.20	0.21	–0.03	0.15	–0.04	0.13	–0.04	0.22	0.00	0.25	–0.03	0.24	0.05	0.26
λ_9,1_	1.00	–0.03	0.09	–0.16	0.17	–0.01	0.20	0.05	0.23	–0.09	0.23	0.08	0.26	–0.08	0.27	0.00	0.23
λ_10,1_	0.96	0.00	0.07	–0.14	0.15	–0.01	0.15	0.06	0.14	–0.03	0.17	0.07	0.17	–0.04	0.10	–0.02	0.11
λ_6,2_	2.16	–0.01	0.24	–0.42	0.48	0.07	0.32	–0.72	0.76	0.20	0.51	–0.79	0.82	0.00	0.48	–0.70	0.81
λ_7,2_	2.14	0.07	0.19	–0.29	0.32	–0.13	0.39	–0.78	0.81	0.14	0.39	–0.58	0.66	0.24	0.40	–0.47	0.54
λ_8,2_	1.98	0.09	0.20	–0.28	0.31	–0.03	0.22	–0.56	0.59	0.13	0.43	–0.51	0.61	0.26	0.39	–0.55	0.63
λ_9,2_	2.05	0.08	0.15	–0.33	0.35	0.12	0.52	–0.44	0.63	0.20	0.34	–0.67	0.76	0.27	0.49	–0.38	0.51
λ_10,2_	2.12	–0.02	0.21	–0.39	0.43	0.10	0.27	–0.52	0.59	0.19	0.28	–0.63	0.64	0.05	0.24	–0.55	0.59
η_1_	0.57	–0.01	0.15			–0.01	0.18			0.09	0.17			–0.02	0.11		
η_2_	0.59	0.01	0.13			0.05	0.17			0.11	0.18			–0.04	0.15		
η_3_	0.70	0.04	0.16			0.04	0.17			0.12	0.18			–0.02	0.13		
η_4_	1.83	0.00	0.17			0.05	0.15			0.10	0.18			–0.03	0.13		
η_5_	–0.50	–0.04	0.16			–0.03	0.14			0.10	0.20			–0.07	0.18		
η_6_	–0.56	0.03	0.16			–0.06	0.17			0.08	0.17			–0.09	0.15		
η_7_	–0.10	0.01	0.18			0.03	0.23			0.09	0.18			–0.04	0.17		
η_8_	–1.05	0.01	0.17			0.07	0.17			0.08	0.17			–0.03	0.17		
η_9_	0.55	0.04	0.15			0.04	0.13			0.10	0.19			–0.04	0.14		
η_10_	–2.03	0.02	0.19			0.10	0.24			0.16	0.21			0.04	0.14		
**Profile recovery**																	
Minimum		96.41		94.27		68.80		59.83		67.44		58.61		68.42		62.40	
Maximum		99.15		97.00		75.67		67.85		73.22		65.24		77.21		69.83	
Mean		97.58		95.78		71.02		63.50		70.14		62.16		72.62		66.20	
*SD*		0.66		0.64		2.14		2.77		1.45		1.83		2.89		2.29	

Focusing on results of the facets-CDM model, the profile recovery rates ranged from 67 to 69% in the three incomplete designs, where each item was rated by three raters. Such findings indicated that if one wishes to obtain a mean profile recovery rate of 70% from ten dichotomous items measuring five attributes, each item should be judged by three raters (i.e., each ratee received 30 scores). Moreover, as indicative by the results of the completely crossed design, if each item is judged by ten raters (i.e., each ratee received 100 scores), the mean profile recovery rate could be as high as 98%.

When rater effects were ignored and the standard CDM was fit, the RMSE values became larger, ranging from 0.14 to 0.62 (*M* = 0.34) in the completely crossed design, from 0.07 to 0.81 (*M* = 0.34) in the balanced incomplete design, from 0.06 to 0.84 (*M* = 0.37) in the unbalanced incomplete design, and from 0.08 to 0.81 (*M* = 0.35) in the random design. The mean recovery rate across profiles was 95.78% in the completely crossed design, 63.50% in the balanced incomplete design, 62.16% in the unbalanced incomplete design, and 66.20% in the random design. Therefore, as expected, the parameter estimation in the standard CDM was worse than those in the facets-CDM. With respect to the recovery of the latent profile, both models yielded a higher recovery rate in the complete design than incomplete designs. This was because in the facets framework, when there are more raters, the measurements are more precise. In the complete design, these two models yielded almost identical recovery rates, which was because the mean rater effect was constrained at zero and thus canceled out. In the incomplete design, the mean rater effect was not canceled out, so the facets model consistently yielded a higher recovery rate (6–8% improvement) than the standard model.

## Simulation Study II: HRM-CDM

### Design and Analysis

The settings were identical to those in simulation study I except only the completely crossed design was adopted and each ratee was judged by three or six raters. The (saturated) HRM-CDM (Equation 17), given the latent category, was used at the second step. At the first step, ϕ_*r*_ and ψ_r_ were fixed at 0 and 0.5, respectively, for all raters. Both the data-generating HRM-CDM and the standard CDM (without considering rater effects) were fit to the simulated data. In the standard CDM, multiple ratings given to the same item response were treated as independent responses. For example, the three sets of ratings given by three raters were analyzed as if the test was answered by three virtual examinees. Then, the posterior probability for each latent attribute (or latent profile) was averaged across the three virtual examinees to represent the examinee’s final estimate. Like in simulation study I, it was expected that the more the raters (the more the data points), the better the parameter estimation and recovery rates. Further, when the standard CDM was fit, the parameter estimation and recovery rates would be poor because the rater effects were not considered.

### Results

Detailed results for individual parameters are not presented due to space constraints but available on request. When the HRM-CDM was fit, the resulting RMSE values ranged from 0.11 to 0.67 (*M* = 0.35) and from 0.08 to 0.43 (*M* = 0.25) for three and six raters, respectively; the mean profile recovery rate was 61.12 and 79.12% for three and six raters, respectively. It appeared that the more the data points the better the parameter estimation and recovery rates when the HRM-CDM was fit. If one wishes to obtain a mean profile recovery rate of 80% from 10 dichotomous items measuring five attributes, it can be found from this simulation study that each item should be judged by six raters (i.e., each ratee received 60 scores). If each item is judged by only three raters (i.e., each ratee received 30 scores), the mean profile recovery rate could be as low as 60%. When the standard CDM was fit, the RMSE values ranged from 0.25 to 0.91 (*M* = 0.57) and from 0.08 to 0.46 (*M* = 0.30) for three and six raters, respectively; the mean profile recovery rate was 56.34 and 70.84% for three and six raters, respectively. Taken together, as anticipated, ignoring rater effects by fitting the standard CDM would yield poor parameter estimation and profile recovery, and the fewer the raters, the worse the parameter and profile recovery. As for the recovery of latent profiles, the HRM-CDM outperformed the standard model, and its superiority (5–10% improvement) was more obvious when more raters were included.

A comparison between the facets-CDM and HRM-CDM revealed that the parameter estimation and profile recovery were better in the former than in the latter. This was mainly because each data point contributed to the parameter estimation directly in the facets-CDM, whereas the scores given by raters provided information about the latent category, which then provided information about the ratee and item parameters in the HRM-CDM. The corresponding JAGS codes for the facets-CDM and HRM-CDM are presented in Appendix.

## Real Data Application

The empirical study involved a total of 287 university students, each producing one academic essay in English, which was judged by one or two teachers (out of nine) against a 52-item checklist. The checklist was developed on the basis of the Empirical Descriptor-based Diagnostic Checklist (Kim, 2011). Each item of the checklist was rated on a binary scale, where 1 = correct, 0 = incorrect. The 52 items aimed to measure six latent attributes of academic writing, namely, content, organization, grammar, vocabulary, conventions of the academic genre, and mechanics. The Q-matrix of the 52 items is shown in Table 4. The data matrix was three-dimensional: 287 examinees by 52 items by 9 raters. Because each item on the diagnostic checklist represented a concrete descriptor of the desirable quality of writing (e.g., item 4 “the essay contains a clear thesis statement”), the scoring rubrics were clear and simple for the raters to follow. Thus, the HRM framework appeared to be preferable to the facets approach. For completeness and illustrative simplicity, three models were fitted using JAGS, including (a) the standard DINA model, in which the ratings from raters were treated as responses to virtual items with identical item parameters; (b) the facet-DINA model; (c) the HRM-DINA model. A normal prior with mean zero and standard deviation four was specified for all item parameters across the three models, except that a log-normal distribution with mean zero and standard deviation four was specified for the variability parameter (ψ_r_) in the HRM-DINA. For the facets-DINA model, a normal distribution with mean zero and standard deviation one was specified for rater parameters, and the mean severity across raters was fixed at zero for model identification. In the HRM-DINA model, the prior distributions for ϕ_*r*_ and ψ_r_ were set as ϕ_r_∼*N*(0,1) and log⁡(ψ_r_)∼*N*(0,4), respectively.

**TABLE 4:** Q-matrix of the 52 criteria in the empirical example.

	Attribute							Attribute					
Item	1	2	3	4	5	6	Item	1	2	3	4	5	6
1	1	1	1	1	1	0	27	0	0	1	0	0	0
2	1	0	0	0	0	0	28	0	0	1	0	0	0
3	1	0	0	0	0	0	29	0	0	1	0	0	0
4	0	1	1	1	0	0	30	0	0	1	0	0	0
5	0	1	1	1	0	0	31	0	0	1	0	0	1
6	1	0	1	1	1	0	32	0	0	1	0	0	1
7	1	1	1	1	0	0	33	0	0	1	0	0	0
8	1	1	0	0	0	0	34	0	0	1	0	0	0
9	1	1	0	0	0	0	35	0	0	1	0	0	0
10	1	1	0	0	0	0	36	0	0	1	1	0	0
11	1	0	0	1	0	0	37	0	0	1	0	0	0
12	1	1	0	1	0	0	38	0	0	1	0	0	0
13	1	0	0	0	0	0	39	0	0	1	1	0	0
14	0	1	0	0	1	0	40	0	0	1	1	0	0
15	0	1	0	0	0	0	41	0	0	0	1	0	0
16	0	1	0	0	0	0	42	0	0	0	1	0	0
17	0	1	0	0	0	0	43	0	0	0	1	0	0
18	0	1	0	0	0	0	44	0	0	0	1	0	0
19	0	1	0	0	0	0	45	0	0	1	1	0	0
20	0	1	0	0	0	0	46	0	0	0	1	0	1
21	0	1	1	1	0	0	47	0	0	0	1	0	1
22	0	1	1	1	0	0	48	0	0	1	0	0	1
23	0	1	1	1	0	0	49	0	0	0	0	0	1
24	0	1	1	1	0	0	50	0	0	0	0	0	1
25	0	1	0	0	0	0	51	0	0	1	1	1	0
26	0	0	1	0	0	0	52	0	0	1	1	1	0

*1s mean the attributes are required, and 0s mean the attributes are not required.*

Table 5 displays the means and standard deviations of the ratings on the 52 descriptors given by the nine raters. Rater 1 gave the lowest mean score (*M* = 0.41), whereas rater 8 gave the highest (*M* = 0.84). For model comparison, Table 6 presents the posterior predictive *p*-values (Gelman et al., 1996) of the Bayesian chi-square statistic, Akaike’s information criterion (AIC), and Bayesian information criterion (BIC) for the three models. The *p*-values suggested all models had a good fit. Both AIC and BIC indicated that the HRM-DINA model was the best-fitting model. Table 7 lists the estimates for the guessing and slip parameters for the three models. The standard DINA model and the facets-DINA model produced very similar estimates. In comparison, the HRM-DINA model yielded smaller estimates for the guessing parameters and larger estimates for the slip parameters than the other two models.

**TABLE 5:** Means and standard deviations for raters’ scorings across all indicators in the empirical example.

Rater	1	2	3	4	5	6	7	8	9
Mean	0.41	0.74	0.68	0.68	0.57	0.58	0.55	0.84	0.59
*SD*	0.28	0.26	0.29	0.26	0.28	0.31	0.27	0.18	0.33

**TABLE 6:** Model fit statistics of the three models in the empirical example.

Model	*ppp*	AIC	BIC
DINA	0.36	17004	17625
Facets DINA	0.44	16690	17331
HRM DINA	0.56	10490	11167

*ppp, posterior predictive p-value; AIC, Akaike’s information criterion; BIC, Bayesian information criterion.*

**TABLE 7:** Estimates for the guessing and slip parameters yielded by the three models in the empirical example.

Item	Guessing			Slip		
	DINA	Facets-DINA	HRM-DINA	DINA	Facets-DINA	HRM-DINA
1	0.49	0.49	0.48	0.03	0.02	0.00
2	0.47	0.47	0.42	0.02	0.03	0.00
3	0.49	0.49	0.49	0.09	0.08	0.12
4	0.46	0.46	0.46	0.05	0.06	0.00
5	0.24	0.30	0.12	0.34	0.36	0.43
6	0.50	0.49	0.49	0.05	0.00	0.00
7	0.16	0.17	0.00	0.52	0.50	0.70
8	0.09	0.11	0.00	0.18	0.20	0.23
9	0.38	0.41	0.28	0.01	0.04	0.00
10	0.31	0.31	0.14	0.23	0.21	0.30
11	0.18	0.17	0.01	0.26	0.27	0.31
12	0.46	0.46	0.38	0.07	0.07	0.01
13	0.48	0.48	0.43	0.08	0.08	0.06
14	0.49	0.49	0.49	0.05	0.00	0.00
15	0.15	0.14	0.00	0.45	0.44	0.56
16	0.48	0.48	0.48	0.08	0.08	0.06
17	0.47	0.47	0.46	0.15	0.15	0.17
18	0.26	0.41	0.23	0.23	0.25	0.31
19	0.46	0.45	0.44	0.15	0.13	0.16
20	0.38	0.24	0.31	0.10	0.07	0.07
21	0.25	0.16	0.09	0.31	0.22	0.13
22	0.49	0.48	0.48	0.12	0.06	0.01
23	0.50	0.49	0.49	0.01	0.01	0.00
24	0.48	0.48	0.48	0.11	0.13	0.06
25	0.45	0.46	0.45	0.07	0.08	0.05
26	0.07	0.12	0.00	0.79	0.81	1.00
27	0.31	0.33	0.02	0.66	0.67	0.96
28	0.40	0.47	0.40	0.15	0.18	0.19
29	0.47	0.47	0.46	0.04	0.05	0.03
30	0.44	0.37	0.29	0.31	0.26	0.39
31	0.39	0.36	0.19	0.45	0.42	0.59
32	0.30	0.42	0.25	0.24	0.26	0.31
33	0.18	0.10	0.02	0.37	0.32	0.41
34	0.08	0.15	0.01	0.54	0.56	0.77
35	0.42	0.47	0.33	0.21	0.24	0.19
36	0.23	0.34	0.06	0.25	0.26	0.27
37	0.45	0.47	0.42	0.11	0.12	0.09
38	0.13	0.17	0.01	0.56	0.59	0.70
39	0.48	0.48	0.48	0.05	0.04	0.00
40	0.00	0.01	0.00	0.93	0.95	1.00
41	0.01	0.07	0.00	0.54	0.58	0.88
42	0.37	0.35	0.39	0.26	0.24	0.41
43	0.23	0.33	0.12	0.28	0.30	0.34
44	0.21	0.33	0.05	0.33	0.38	0.49
45	0.19	0.21	0.02	0.07	0.18	0.02
46	0.28	0.27	0.09	0.02	0.11	0.00
47	0.49	0.49	0.48	0.04	0.07	0.01
48	0.49	0.49	0.49	0.01	0.02	0.00
49	0.49	0.49	0.49	0.00	0.02	0.00
50	0.04	0.24	0.00	0.43	0.69	0.95
51	0.33	0.47	0.24	0.13	0.31	0.37
52	0.03	0.30	0.00	0.40	0.66	0.92

Estimates for rater severity (ϕ) and variability (ψ) under the HRM-DINA model are presented in Table 8. Among the 9 raters, rater 1 was the most severe, followed by rater 7, while the others had severity measures around 0. Both rater 1 and rater 7 tended to assign ratings lower than what the ratees deserved (their severity parameters were positive). Furthermore, the estimates for rater variability ranged from 0.37 (rater 1) to 1.28 (rater 8), suggesting the raters exhibited moderate to high variability in their ratings.

**TABLE 8:** Rater severity and variability yielded from the HRM DINA model in the empirical example.

Rater	1	2	3	4	5	6	7	8	9
Severity	0.40	0.02	0.01	0.02	0.01	0.02	0.24	0.04	0.02
SE	0.02	0.05	0.06	0.06	0.05	0.06	0.00	0.07	0.02
Variability	0.37	0.84	0.69	0.70	0.53	0.52	0.76	1.28	0.49
SE	0.01	0.05	0.04	0.04	0.03	0.03	0.04	0.10	0.02

Regarding the attribute estimates, the mastery probabilities of the six attributes were 50, 77, 76, 69, 63, and 73% for the standard DINA model, 53, 81, 77, 78, 83, and 79% for the facets-DINA model, and 50, 71, 68, 66, 75, and 74% for the HRM-DINA model. Among the 287 students, 77 students (27%) resulted in identical profile estimates with the three models, indicating moderate similarity on profile estimates across the three models.

To show the effects of ignoring rater effects, we picked up five students from the real data. For the selected cases, they were rated either by Rater 1, who tended to be the most severe, or by Rater 8, who tended to be most lenient. The differences between observed and fair scores (the expected score given the item and person parameters) are shown in Table 9. If one wants to admit students to some program according to their observed (raw) scores, then the ordering will be no. 69, 230, 21, 30, and 23, respectively. After taking into consideration of the rater effect by fitting the facets-DINA, we have fair score for each student. Now, if one wants to admit the five students according to the fair scores, then the ordering will be student no. 21, 69, 23, 230, and 30, respectively. Obviously, the two rank orderings were very different, which was because the former did not consider rater effect.

**TABLE 9:** Fair scores and observed scores for selected cases in the real data.

Student index	Estimated profile	Rater	Observed scores	Fair scores	Difference
21	1,1,1,1,1,0	1	23	40	−17
23	0,1,1,1,1,1	1	13	36	−23
30	0,0,0,1,0,0	1	15	22	−7
69	1,0,1,1,0,1	8	44	38	6
230	1,1,1,1,0,1	8	42	33	9

*Estimated profiles were obtained by fitting facets-DINA model. Fair scores were calculated by DINA model with given person profile and item parameters.*

## Conclusion and Discussion

Rater effects on CR items have been investigated extensively within the frameworks of IRT-facets and IRT-HRM, but not within those of CDMs. In this study, we adopted the facets and HRM frameworks and used the LCDM as a template to create new facets-CDM and HRM-CDM to accommodate rater effects. We also conducted simulations to evaluate parameter recovery of the new models under various conditions. Results indicate that model parameters could be estimated fairly well with JAGS package in R. Implications and applications of the new models were demonstrated with an empirical study that assessed English academic essays by university students. In the empirical study, the scales of the guessing and slip parameters for standard DINA and facets-DINA models were very similar, but they were very different from those for the HRM-DINA model, which was mainly because the HRM-DINA model was formed in a very different way from the other two models. Under the HRM-DINA model, among the 9 raters, raters 1 and 7 were the most severe. In addition, the rater variability ranged from 0.37 to 1.28, suggesting a moderate to high variability in their ratings.

Several limitations of the current study should be acknowledged. First, despite our efforts in testing the new models under different rating designs, the simulated conditions of the present study is not comprehensive. Future studies should be conducted to evaluate the performance of the new models under more comprehensive conditions, such as different test lengths, sample sizes, rater sizes, and rater designs. Second, a good CDM test depends on the quality of the Q-matrix (Lim and Drasgow, 2017). In this study, only one Q-matrix was used. How the facets- and HRM-CDMs perform with different Q-matrices needs further investigation. Third, like other simulation studies of CDMs, the data were analyzed with the data-generating models without looking to other potential sources of model-data misfit, such as mis-specification of the model or Q-matrix. Sensitivity analysis of the new models is warranted. Finally, the long computing time for MCMC methods may be a concern for potential users, especially for large scale data sets with long test length and large sample size. Future attempts are needed to develop more efficient and effective estimation programs.

Future studies can also be conducted to extend the new facets- and HRM-CDMs. For instance, the linear combination of parameters in the facets- or HRM-CDMs can be extended to account for interactions among facets (Jin and Wang, 2017). It is feasible to develop explanatory facets- or HRM-CDMs by incorporating covariates (e.g., gender or language background) to account for the variations in rater effects (Ayers et al., 2013). Large-scale educational testing services often recruit a large number of raters (e.g., hundreds of raters), where it would be more efficient to treat rater severity as a random effect following some distributions (e.g., normal distributions). Finally, this study focuses on dichotomous items because the majority of existing CDMs focus on binary data. New facets- or HRM-CDMs can be developed to accommodate polytomous CR items, just as CDMs has been extended to accommodate polytomous items, as shown in Equations 11, 16, and 19, or those in the literature (Hansen, 2013; Ma and de la Torre, 2016).

## Data Availability Statement

The datasets generated for this study are available on request to the corresponding author.

## Ethics Statement

The studies involving human participants were reviewed and approved by the Human Research Ethics Committee of The Education University of Hong Kong. The patients/participants provided their written informed consent to participate in this study.

## Author Contributions

XL and W-CW conceived and designed the study, performed the simulations and analyses, interpreted the results, and wrote the manuscript. QX contributed the empirical data, provided critical comments on the study, and edited the whole manuscript. All authors provided the final approval of the version to publish.

## Conflict of Interest

The authors declare that the research was conducted in the absence of any commercial or financial relationships that could be construed as a potential conflict of interest.

## References

[B1] Ayers E. Rabe-Hesketh S. Nugent R. (2013). Incorporating student covariates in cognitive diagnosis models. *J. Classif.* 30 195–224. 10.1007/s00357-013-9130-y

[B2] Casabianca J. M. Junker B. W. Nieto R. Bond M. A. (2017). A hierarchical rater model for longitudinal data. *Multivar. Behav. Res.* 52 576–592. 10.1080/00273171.2017.1342202 28846050

[B3] Chiu C. Seo M. (2009). Cluster analysis for cognitive diagnosis: an application to the 2001 PIRLS reading assessment. *IERI Monogr. Ser. Issues Methodol. Large Scale Assess.* 2 137–159.

[B4] Chiu C.-Y. Douglas J. (2013). A nonparametric approach to cognitive diagnosis by proximity to ideal response patterns. *J. Classif.* 30 225–250. 10.1007/s00357-013-9132-9

[B5] Chiu C.-Y. Douglas J. A. Li X. (2009). Cluster analysis for cognitive diagnosis: theory and applications. *Psychometrika* 74 633–665. 10.1007/s11336-009-9125-0 25496248

[B6] de la Torre J. (2011). The generalized DINA model framework. *Psychometrika* 76 179–199. 10.1007/s11336-011-9207-7

[B7] de la Torre J. Douglas J. A. (2004). Higher-order latent trait models for cognitive diagnosis. *Psychometrika* 69 333–353. 10.1007/BF02295640

[B8] De la Torre J. Hong Y. Deng W. (2010). Factors affecting the item parameter estimation and classification accuracy of the DINA model. *J. Educ. Meas.* 47 227–249. 10.1111/j.1745-3984.2010.00110.x

[B9] DeCarlo L. T. Kim Y. K. Johnson M. S. (2011). A hierarchical rater model for constructed responses, with a signal detection rater model. *J. Educ. Meas.* 48 333–356. 10.1111/j.1745-3984.2011.00143.x

[B10] Engelhard G. Jr. (1994). Examining rater errors in the assessment of written composition with a many-faceted Rasch model. *J. Educ. Meas.* 31 93–112. 10.1111/j.1745-3984.1994.tb00436.x

[B11] Engelhard G. (1996). Evaluating rater accuracy in performance assessments. *J. Educ. Meas.* 33 56–70. 10.1111/j.1745-3984.1996.tb00479.x

[B12] Gelman A. Meng X. L. Stern H. (1996). Posterior predictive assessment of model fitness via realized discrepancies. *Stat. Sin.* 6 733–807.

[B13] Gelman A. Rubin D. B. (1992). Inference from iterative simulation using multiple sequences. *Stat. Sci.* 7 457–511. 10.1214/ss/1177011136

[B14] Haertel E. H. (1989). Using restricted latent class models to map the skill structure of achievement items. *J. Educ. Meas.* 26 301–323. 10.1111/j.1745-3984.1989.tb00336.x

[B15] Hansen M. (2013). *Hierarchical Item Response Models for Cognitive Diagnosis.* Doctoral dissertation, University of California, Los Angeles, CA.

[B16] Hansen M. Cai L. (2013). Abstract: a hierarchical item response model for cognitive diagnosis. *Multivar. Behav. Res.* 48:158. 10.1080/00273171.2012.748372 26789220

[B17] Hartz S. M. (2002). *A Bayesian Framework for the Unified Model for Assessing Cognitive Abilities: Blending Theory With Practicality.* Doctoral dissertation, University of Illinois, Urbana-Champaign, IL.

[B18] Henson R. Templin J. Willse J. (2009). Defining a family of cognitive diagnosis models using log-linear models with latent variables. *Psychometrika* 74 191–210. 10.1007/s11336-008-9089-5

[B19] Huang H.-Y. Wang W.-C. (2014). The random-effect DINA model. *J. Educ. Meas.* 51, 75–97. 10.1111/jedm.12035

[B20] Jin K. Y. Wang W. C. (2017). Assessment of differential rater functioning in latent classes with new mixture facets models. *Multivar. Behav. Res.* 52 391–402. 10.1080/00273171.2017.1299615 28328280

[B21] Junker B. W. Sijtsma K. (2001). Cognitive assessment models with few assumptions, and connections with nonparametric item response theory. *Appl. Psychol. Meas.* 25 258–272. 10.1177/01466210122032064

[B22] Kim Y. H. (2011). Diagnosing EAP writing ability using the reduced reparameterized unified model. *Lang. Test.* 28 509–541. 10.1177/0265532211400860

[B23] Li X. Wang W.-C. (2015). Assessment of differential item functioning under cognitive diagnosis models: the DINA model example. *J. Educ. Meas.* 52 28–54. 10.1111/jedm.12061

[B24] Lim Y. S. Drasgow F. (2017). Nonparametric calibration of item-by-attribute matrix in cognitive diagnosis. *Multivar. Behav. Res.* 52 562–575. 10.1080/00273171.2017.1341829 28715230

[B25] Linacre J. M. (1989). *Many-Facet Rasch Measurement.* Chicago, IL: MESA Press.

[B26] Ma W. de la Torre J. (2016). A sequential cognitive diagnosis model for polytomous responses. *Br. J. Math. Stat. Psychol.* 69 253–275. 10.1111/bmsp.12070 27317397

[B27] Masters G. N. (1982). A rasch model for partial credit scoring. *Psychometrika* 47 149–174. 10.1007/BF02296272

[B28] Myford C. M. Wolfe E. W. (2003). Detecting and measuring rater effects using many-facet Rasch measurement: Part I. *J. Appl. Meas.* 4 386–422. 14523257

[B29] Myford C. M. Wolfe E. W. (2004). Detecting and measuring rater effects using many-facet Rasch measurement: Part II. *J. Appl. Meas.* 5 189–227. 15064538

[B30] Patz R. J. Junker B. W. Johnson M. S. Mariano L. T. (2002). The hierarchical rater model for rated test items and its application to large scale educational assessment data. *J. Educ. Behav. Stat.* 27 341–384. 10.3102/10769986027004341

[B31] Plummer M. (2015). *coda (R Package Version 0.18-1, pp. 1–45).* Vienna: The Comprehensive R Archive Network.

[B32] R Core Team (2016). *R: A Language and Environment For Statistical Computing.* Vienna: The R Foundation for Statistical Computing.

[B33] Su Y. Yajima M. (2015). *R2jags (R Package Version 0.5-7, pp. 1–12).* Vienna: The Comprehensive R Archive Network.

[B34] Tatsuoka K. (1985). A probabilistic model for diagnosing misconceptions in the pattern classification approach. *J. Educ. Stat.* 12 55–73. 10.3102/10769986010001055

[B35] Tatsuoka K. Corter J. Tatsuoka C. (2004). Patterns of diagnosed mathematical content an process skills in TIMSS-R across a sample of 20 countries. *Am. Educ. Res. J.* 41 901–926. 10.3102/00028312041004901

[B36] Templin J. (2004). *Generalized Linear Mixed Proficiency Models for Cognitive Diagnosis.* Ph.D. dissertation, University of Illinois, Urbana-Champaign, IL.

[B37] Templin J. L. Bradshaw L. (2013). Measuring the reliability of diagnostic classification model examinee estimates. *J. Classif.* 30 251–275. 10.1007/s00357-013-9129-4 20484592

[B38] Templin J. L. Henson R. A. (2006). Measurement of psychological disorders using cognitive diagnosis models. *Psychol. Methods* 11 287–305. 10.1037/1082-989X.11.3.287 16953706

[B39] von Davier M. (2008). A general diagnostic model applied to language testing data. *Br. J. Math. Stat. Psychol.* 61 287–307. 10.1002/j.2333-8504.2005.tb01993.x 17535481

[B40] Wang W.-C. Liu C.-Y. (2007). Formulation and application of the generalized multilevel facets model. *Educ. Psychol. Meas.* 67 583–605. 10.1177/0013164406296974

[B41] Wang W.-C. Wilson M. (2005). Exploring local item dependence using a random-effects facet model. *Appl. Psychol. Meas.* 29 296–318. 10.1177/0146621605276281

[B42] Xu X. von Davier M. (2008). *Fitting the Structured General Diagnostic Model to NAEP Data (Research Report No. 08-27).* Princeton, NJ: Educational Testing Service.

[B43] Zhan P. Jiao H. Man K. Wang L. (2019). Using JAGS for bayesian cognitive diagnosis modeling: a tutorial. *J. Educ. Behav. Stat.* 44 473–503. 10.3102/1076998619826040 27193366

